# Effect of proprotein convertase subtilisin/kexin type 9 inhibition on cancer events: A pooled, post hoc, competing risk analysis of alirocumab clinical trials

**DOI:** 10.1002/cam4.6310

**Published:** 2023-07-17

**Authors:** Kusha A. Mohammadi, Taylor Brackin, Gregory G. Schwartz, Philippe Gabriel Steg, Michael Szarek, Garen Manvelian, Robert Pordy, Sergio Fazio, Gregory P. Geba

**Affiliations:** ^1^ Regeneron Pharmaceuticals, Inc. Tarrytown New York USA; ^2^ University of Colorado School of Medicine Aurora Colorado USA; ^3^ Université Paris‐Cité Paris France; ^4^ FACT (French Alliance for Cardiovascular Trials) INSERM U1148 Paris France; ^5^ Assistance Publique‐Hôpitaux de Paris Hôpital Bichat Paris France; ^6^ State University of New York Downstate School of Public Health Brooklyn New York USA; ^7^ CPC Clinical Research and Division of Cardiology University of Colorado School of Medicine Aurora Colorado USA

**Keywords:** alirocumab, cholesterol, incident cancer, LDL‐C, PCSK9 inhibitor, serum lipids

## Abstract

**Objective:**

Assess the risk of new and worsening cancer events among participants who received the lipid‐lowering therapy alirocumab, a proprotein convertase subtilisin/kexin type 9 inhibitor.

**Design:**

Pooled post hoc analysis.

**Setting:**

Six phase 3 or phase 4 placebo‐controlled randomised trials with alirocumab.

**Participants:**

A total of 24,070 patients from the safety population with complete dosing data (alirocumab, *n* = 12,533; placebo, *n* = 11,537).

**Intervention:**

Alirocumab 75 mg, alirocumab 150 mg, alirocumab 75 mg increasing to 150 mg if low‐density lipoprotein cholesterol <50 mg/dL not achieved, or placebo, all every 2 weeks. All participants received background high‐intensity or maximum‐tolerated statin therapy.

**Outcomes and Measures:**

The first new or worsening incident cancer events were assessed during the treatment‐emergent adverse event period. Four outcomes were evaluated: any‐neoplasm, malignant neoplasms, broad definition of hormone‐sensitive cancers, and stricter definition of hormone‐sensitive cancers. Sub‐distribution hazard ratios and 95% confidence intervals (CIs) were estimated using a competing risk framework, with death as a competing risk.

**Results:**

Considering both treatment arms in aggregate, 969 (4.03%), 779 (3.24%), 178 (0.74%) and 167 (0.69%) patients developed any neoplasm, malignant neoplasms, broad definition of hormone‐sensitive cancer and strict definition of hormone‐sensitive cancer events, respectively. There was no significant difference in the risk of having any neoplasm in the alirocumab versus the placebo group (sub‐distribution hazards ratio [95% CI], 0.93 [0.82–1.1]; *p* = 0.28). A nominally lower risk of having any neoplasms with alirocumab was observed among subjects aged ≥64 years (sub‐distribution hazards ratio 0.83; 95% CI, 0.70–0.99).

**Conclusions:**

Intensive low‐density lipoprotein cholesterol lowering with a proprotein convertase subtilisin/kexin type 9 inhibitor combined with statin does not appear to increase the risk of new or worsening cancer events.

## Graphical abstract

The risk of new and worsening cancer events was assessed among participants who received the proprotein convertase subtilisin/kexin type 9 inhibitor alirocumab combined with a statin. Our results demonstrated that intensive low‐density lipoprotein cholesterol lowering does not appear to increase the risk of new or worsening cancer events.

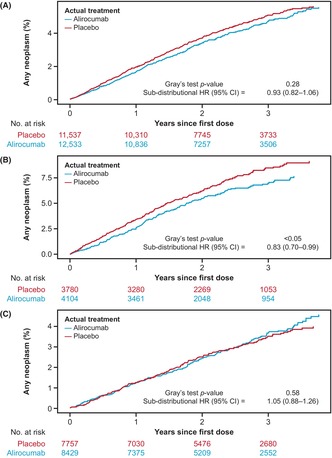

## 1 INTRODUCTION

Cancer is a major cause of mortality worldwide and is second only to heart disease as the most common cause of death in the USA.^1^, ^2^, ^3^ Cholesterol metabolism is possibly associated with the initiation and progression of cancer given its role in multiple cellular activities, including cell proliferation.^4^, ^5^ Furthermore, sex hormones, which are dependent on cholesterol for their synthesis, influence, among others, colon, prostate and breast cancers.^6^, ^7^, ^8^

Cellular cholesterol synthesis is tightly regulated by the rate‐limiting step of 3‐hydroxy‐3‐methyl‐glutaryl‐coenzyme A (HMG‐CoA) reductase.^9^ Whether statin therapy, which inhibits HMG‐CoA reductase, affects cancer rates remains a topic of discussion.^10^

Epidemiological studies have not provided a clear connection between plasma lipid levels and incidence of cancer. To date, trials of standard lipid‐lowering therapies (mostly statins) have not supported an association of treatment with cancer risk.^11^, ^12^, ^13^, ^14^, ^15^, ^16^, ^17^ A recent literature review summarising evidence from 41 studies found no evidence of association between native (untreated) cholesterol levels and the incidence of hormonally driven cancer.^18^

The addition of newer lipid‐lowering therapies, such as proprotein convertase subtilisin/kexin type 9 (PCSK9) inhibitors, enable patients to achieve much lower levels of low‐density lipoprotein cholesterol (LDL‐C) than with standard therapies alone.^19^, ^20^, ^21^ PCSK9 acts to increase the degradation of low‐density lipoprotein receptors (LDLR),^22^ and PCSK9 blockade therefore increases LDLR levels and removal of LDL‐C from the circulation, leading to intensive LDL‐C lowering. Due to the integral role of PCSK9 in lipid metabolism, PCSK9 function and its inhibition could potentially influence cancer pathophysiology.^23^, ^24^, ^25^ A systematic review of trials of PCSK9‐inhibiting antibodies to quantify the incidence of cancer associated with their use reported an odds ratio of 0.88 (95% confidence interval [CI], 0.61–1.18) and concluded there was no effect on cancer diagnosis.^26^

A recent large Mendelian randomization study showed that gene variants diminishing HMG‐CoA reductase activity (the mechanism of action of statins) were associated with a lower incidence of cancer. However, this protective effect was not observed for loss‐of‐function genetic variants of *PCSK9*,^27^ suggesting that PCSK9 inhibitors and statins may have separate and distinct effects on cancer rates.

Alirocumab, one of the two commercially available monoclonal antibodies that binds PCSK9, preventing it from binding to the LDLR, demonstrated up to 61% reduction in LDL‐C levels in patients with hypercholesterolaemia receiving maximally tolerated statin therapy during the phase 3 ODYSSEY clinical trial programme.^28^, ^29^, ^30^, ^31^ The LDL‐C lowering with alirocumab was associated with significantly fewer cardiovascular events and lower all‐cause mortality in the large ODYSSEY OUTCOMES trial.^19^, ^32^

The aim of the present analysis was to evaluate the risk of new and worsening cancer events in subjects randomised to alirocumab compared with placebo through a rigorous adjudication process of individual level safety records. In this post hoc analysis of trials in the ODYSSEY programme, we determined incident cancer events among participants in five phase 3 and one phase 4 study.^33^ The risk of most adult cancers is known to increase with increasing age,^34^ and the cohort provided an excellent opportunity to assess cancer incidence in a population at high risk.

## 2 METHODS

### 2.1 Study design

This post hoc analysis assessed the risk of new and worsening cancers among participants who received the PCSK9 inhibitor alirocumab, using pooled data from five phase 3 and one phase 4 randomised, placebo‐controlled clinical trials.^19^, ^28^, ^29^, ^30^, ^31^, ^33^ The trial designs have been reported previously and are summarised in online Figure S1. All studies were conducted in accordance with the Declaration of Helsinki and the International Conference on Harmonisation Guidelines for Good Clinical Practice. All study protocols were approved by the appropriate institutional review board/ethics review committees, and patients provided written informed consent.

For the present analysis, the time from the first dose of study treatment to the first occurrence of each cancer outcome was determined. Analyses were performed on patients with complete first and last dose dates, with grouping based on the treatment received. Patients from the safety populations of each of the component trials were included, defined as those who received at least one dose or part of a dose of treatment. New or worsening cancer events were assessed during the treatment‐emergent adverse event (TEAE) period, defined for each patient as the interval between the first study dose and the last study dose +70 days (based on five half‐lives), or until death if that occurred earlier.

Outcomes were determined through blinded medical review of adverse event records within the System Organ Class (SOC) of ‘neoplasms benign, malignant, and unspecified (including cysts and polyps)’, with outcomes adjudicated by clinician reviewers unaware of randomised treatment, lipid levels, or clinical variables in the database. The any‐neoplasm outcome was a composite of benign, unspecified and malignant neoplasms. Three additional outcomes were considered: malignant neoplasms, broad definition of hormone‐sensitive cancers and strict definition of hormone‐sensitive cancers. The broad definition of hormone‐sensitive cancers consisted of breast, prostate, uterine, ovarian, pancreas, gallbladder, liver, colorectal and gastrointestinal events. Hormone‐sensitive cancers in the strict definition included breast, prostate, uterine and ovarian events. Stage IV and metastatic cancer events with a start date within 365 days of the patient's first dose date were not included in the analyses. Similarly, stage II and III cancer events with a start date within 90 days of the patient's first dose date were excluded. The exclusionary period for events in patients with stage IV cancer was longer than that for stage II/III cancer because of the lower likelihood that early events in patients with more severe disease were influenced by the trial intervention. Of note, there were only 35 patients with stage IV cancer and reported events within 365 days, and seven patients with stage II/III cancer and reported events within 90 days. To identify prior history of malignancy, the search consisted of similar SOC terms in the medical history database and adjudicated through a blinded review. Worsening events were defined as reported neoplasm events that occurred during the TEAE period in individuals who had cancer by medical history. New events were defined as reported neoplasm in those who had no prior history of malignancy at baseline.

Sub‐distribution hazard ratios (HRs) and 95% CIs were estimated with the use of the Fine–Grey competing‐risk regression model, with death as a competing terminal event and study treatment received as the only model term; *p*‐values were reported with the use of Grey's test. Cumulative incidence figures were truncated when the at‐risk cohort dropped below 15% of baseline. Cause‐specific hazard model and Cox‐proportional hazard model results with log‐rank test *p*‐values are additionally provided. All proportional hazard assumptions were held via a scaled Schoenfeld residuals test. No adjustment for multiplicity was utilised, and all results reflect a nominal alpha‐level of 0.05. Subgroup analyses based on age were performed based on the median age of patients with events. All analyses were done in SAS version 9.4 (SAS Institute Inc., Cary, NC, USA) and R version 4.1.2 (CRAN R Project).

### 2.2 Patient and public involvement

Patients or the public were not involved in the design, or conduct, or reporting, or dissemination plans of our research.

## 3 RESULTS

### 3.1 Participants

This pooled analysis of six alirocumab clinical trials included 24,070 patients from the safety populations with complete dosing data (alirocumab, *n* = 12,533; placebo, *n* = 11,537). The mean duration of the TEAE period for all patients was 2.45 years (median 2.49 years; Q1:Q3 1.65:3.22 years; online Figure S2).

### 3.2 Baseline characteristics of patients

The baseline characteristics for all patients according to treatment received are summarised in Table 1; baseline characteristics by all‐neoplasm outcome are summarised in the online Table S1. No notable differences were observed in relation to age, sex, race, ethnicity, geographic location, body mass index, or baseline lipid levels by treatment arm. Patients who had any neoplasm event were older compared with those without an event (median age of 64 vs. 59 years).

**TABLE 1:** Summary of demographics and baseline characteristics by treatment received.

Characteristic	Placebo (*n* = 11,537)	Alirocumab (*n* = 12,533)	Overall (*N* = 24,070)
Age, median (IQR), year	59.0 (52.0–66.0)	59.0 (52.0–66.0)	59.0 (52.0–66.0)
Sex, *n* (%)			
Male	8318 (72)	8935 (71)	17,253 (72)
Female	3219 (28)	3598 (29)	6817 (28)
Race, *n* (%)			
White	9323 (81)	10,211 (81)	19,534 (81)
Black or African American	368 (3.2)	399 (3.2)	767 (3.2)
Asian	1274 (11)	1299 (10)	2573 (11)
Other	572 (5.0)	624 (5.0)	1196 (5.0)
Weight, median (IQR), kg	82.0 (71.6–93.4)	82.5 (72.0–94.0)	82.2 (71.9–93.9)
BMI, median (IQR)	28.2 (25.4–31.6)	28.3 (25.5–31.6)	28.3 (25.5–31.6)
Smoking status, *n* (%)			
Current	2272 (20)	2279 (18)	4551 (19)
Former or never	7169 (62)	7172 (57)	14,341 (60)
Missing	2096 (18)	3082 (25)	5178 (22)
Drinking status, *n* (%)			
Yes	1841 (16)	1943 (16)	3784 (16)
No	7598 (66)	7507 (60)	15,105 (63)
Missing	2098 (18)	3083 (25)	5181 (22)
Hypertension status, *n* (%)			
Yes	6031 (52)	6197 (49)	12,228 (51)
No	3410 (30)	3254 (26)	6664 (28)
Missing	2096 (18)	3082 (25)	5178 (22)
History of any neoplasm diagnosis, *n* (%)	252 (2.2)	247 (2.0)	499 (2.1)
Region of enrolment, *n* (%)			
Asia	1156 (10)	1170 (9.6)	2326 (9.9)
East Europe	3316 (29)	3485 (28)	6801 (29)
North America	2068 (18)	2385 (19)	4453 (19)
Rest of world	775 (6.9)	832 (6.8)	1607 (6.8)
South America	1430 (13)	1459 (12)	2889 (12)
West Europe	2501 (22)	2906 (24)	5407 (23)
Apolipoprotein A1, median (IQR), mg/dL	134.0 (119.0–150.0)	134.0 (120.0–151.0)	134.0 (119.0–151.0)
Lipoprotein(a), median (IQR), mg/dL	21.3 (6.6–60.7)	21.0 (6.9–60.0)	21.1 (6.8–60.3)
Total cholesterol, median (IQR), mg/dL	164.9 (144.8–191.9)	166.8 (146.0–195.4)	165.6 (145.6–193.4)
Low‐density lipoprotein cholesterol, median (IQR), mg/dL	89.6 (75.0–111.0)	91.5 (76.0–114.0)	90.3 (75.7–112.7)
High‐density lipoprotein cholesterol, median (IQR), mg/dL	43.0 (37.0–51.0)	43.6 (37.1–51.7)	43.2 (37.0–51.4)
Triglycerides, median (IQR), mg/dL	131.0 (95.6–185.8)	131.0 (94.7–184.0)	131.0 (95.0–184.1)
Haemoglobin A1c, median (IQR), %	5.9 (5.5–6.4)	5.8 (5.5–6.4)	5.8 (5.5–6.4)

Abbreviations: BMI, body mass index; IQR, interquartile range.

### 3.3 Incidence of first cancer events

For each outcome evaluated, the incidence and incidence rates for new or worsening cancer events were not significantly different between the alirocumab and placebo groups (Table 2). Across treatment arms, 969 patients (4.03% of total) reported any neoplasms, of whom 472 (3.77%) were in the pooled alirocumab group and 497 (4.31%) were in the placebo group (Table 2). Malignant neoplasms were reported in 2.54% and 2.97% of patients across the alirocumab and placebo arms, respectively. Over the TEAE period, 95 (0.60%) versus 104 (0.72%) patients were identified to have broad definition hormone sensitive cancers in the alirocumab and placebo groups, respectively. Similarly, for strict definition hormone sensitive cancer outcomes, 65 (0.52%) patients were identified in the alirocumab treatment arm, and 71 (0.62%) patients were identified in the placebo arm. The incidence of cancer events broken down by endpoint, clinical trial and treatment arm is summarised in the online Table S2. The breakdown of the components for each outcome by treatment arm is given in the online Table S3.

**TABLE 2:** Summary of cancer incidence by outcome during the treatment‐emergent adverse event period according to treatment group.

Outcome and type	Placebo (*n* = 11,537)			Alirocumab (*n* = 12,523)		
	Total events, *n* ^a^	*n* (%)	Rate per 100 person‐years	Total events, *n* ^a^	*n* (%)	Rate per 100 person‐years
First any neoplasm	732	497 (4.31)	1.73	638	472 (3.77)	1.62
New		454 (3.94)	1.58		440 (3.51)	1.51
Worsening		43 (0.37)	0.15		32 (0.26)	0.11
First malignant neoplasm	525	343 (2.97)	1.18	436	318 (2.54)	1.09
New		306 (2.65)	1.06		296 (2.36)	1.01
Worsening		37 (0.32)	0.13		22 (0.18)	0.08
First hormone sensitive–broad	104	83 (0.72)	0.28	95	75 (0.60)	0.25
New		76 (0.66)	0.26		74 (0.59)	0.25
Worsening		7 (0.06)	0.02		1 (0.01)	0.003
First hormone sensitive–strict	86	71 (0.62)	0.242	81	65 (0.52)	0.220
New		66 (0.57)	0.225		65 (0.52)	0.220
Worsening		5 (0.04)	0.017		0 (0.0)	–

*Note*: Any neoplasm is defined as benign, unspecified, or malignant neoplasm. Malignant neoplasm contains only identified malignant neoplasms. Hormone‐sensitive cancer–broad includes breast, prostate, uterine, ovarian, pancreas, gallbladder, liver, colorectal and gastrointestinal cancers. Hormone sensitive cancer–strict includes breast, prostate, uterine and ovarian cancers.

^a^ Total events refers to all the cancer‐related adverse events that occurred during the clinical trial (i.e., some subjects may have more than one cancer event during the clinical trial).

### 3.4 Cumulative incidence of first cancer events

**FIGURE 1:**
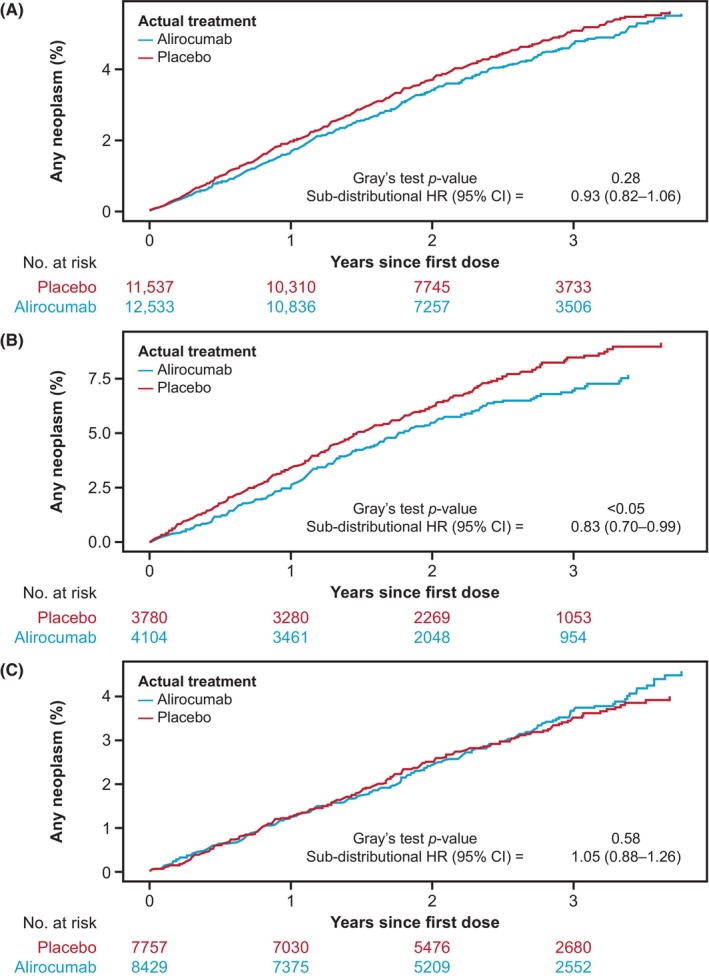
Cumulative incidence using competing risk for time to any neoplasm during the treatment‐emergent adverse event period in (A) the whole population, and among those (B) older or (C) younger than the median age of patients with cancer (64 years). CI, confidence interval; HR, hazard ratio.

The cumulative incidence for having any neoplasm during the TEAE period and in a subgroup analysis by age is shown in Figure 1. There was no significant difference in the risk of having neoplasms for the alirocumab group versus the placebo group for the overall population (sub‐distribution HR [95% CI], 0.93 [0.82–1.10]; nominal *p* = 0.28), or among patients who were older (Figure 1B) or younger (Figure 1C) than the median age (64 years) of those with any neoplasm during the TEAE period. There was a nominally lower risk of having neoplasms among patients aged ≥64 years with a sub‐distribution HR of 0.83 (95% CI, 0.70–0.99), while no difference was observed between treatment groups in those aged <64 years (sub‐distribution HR [95% CI], 1.05 [0.88–1.26]).

When assessing the time to first malignant neoplasm, there was no significant effect of treatment with alirocumab versus placebo (sub‐distribution HR [95% CI], 0.92 [0.9–1.07]; nominal *p* = 0.26; Table 3) in the overall population (online Figure S3A), in those aged ≥64 years (sub‐distribution HR [95% CI], 0.86 [0.70–1.06]; online Figure S3B) or in those aged <64 years (sub‐distribution HR [95% CI], 0.99 [0.79–1.24]; online Figure S3C). The cumulative incidence for hormone‐sensitive cancers, in both the broad and strict definitions, revealed no differences in risk between treatment groups, either for the overall population or for those aged ≥64 or <64 years (online Figure S4 and online Figure S5).

**TABLE 3:** Hazard model results for time to first cancer outcome during the treatment‐emergent adverse event period.

Hazard model type and outcome	Alirocumab versus placebo		
	HR (95% CI)	Model‐based *p* value	Log rank/Grey's test *p* value
Sub‐distributional			
First any neoplasm	0.93 (0.82–1.06)	0.28	0.28
First malignant neoplasm	0.92 (0.79–1.07)	0.26	0.26
First hormone sensitive–broad	0.89 (0.65–1.22)	0.48	0.49
First hormone sensitive–strict	0.90 (0.65–1.27)	0.56	0.56

*Note*: Any neoplasm is defined as benign, unspecified, or malignant neoplasms. Malignant neoplasm contains only identified malignant neoplasms. Hormone‐sensitive cancer–broad includes breast, prostate, uterine, ovarian, pancreas, gallbladder, liver, colorectal and gastrointestinal cancers. Hormone‐sensitive cancer–strict includes breast, prostate, uterine and ovarian cancers. Hazard ratio (95% CI) was based on treatment received as the only model term.

Abbreviations: CI, confidence interval; HR, hazards ratio.

Online Table S4 summarises the results of sensitivity analyses for the overall population using a cause‐specific model and Cox model, all showing no significant effect on the risk for first cancer event in treated subjects with HRs ranging from 0.89 to 0.93 and model‐based *p*‐values ranging from 0.2 to 0.5.

## 4 DISCUSSION

The present analysis was aimed at understanding the relationship between the use of alirocumab, known for its significant LDL‐C lowering effect, and cancer incidence in the setting of the alirocumab clinical trial program through a blinded adjudication process to identify individual cancer events on the individual patient level. We evaluated the risk of new or worsening cancer events in four outcomes among participants of one phase 4, and five phase 3 trials of the ODYSSEY programme (including the ODYSSEY OUTCOMES trial), trials in which all participants also received background high‐intensity or maximum‐tolerated statin therapy. Overall, our results demonstrate no difference in the risk of having any neoplasm as an adverse event between the alirocumab and placebo groups. As expected, patients with a reported cancer event were older than those without. For the older cohort of patients (aged ≥64 years, 64 years being the median age of those with any neoplasm) the risk of having any neoplasm was nominally lower with alirocumab than placebo.

Previous epidemiological studies assessing the effect of statins on the risk of cancer have provided conflicting results, with reports of both a reduced risk and no association, dependent upon cancer type.^14^, ^15^, ^16^, ^17^, ^35^, ^36^, ^37^, ^38^ More recently, a Mendelian randomization study by Carter et al reported that genetic variants reducing the function of *HMGCR* (proxies for statin treatment) were associated with a reduced overall risk of cancer, whereas variants in other genes with influence on lipid metabolism (proxies for other lipid‐lowering therapies) were not.^27^ Statins have pleiotropic effects that may influence cancer progression^27^; they affect isoprenoid synthesis in addition to cholesterol synthesis. Isoprenoids are also thought to influence cancer development via their action as post‐translational modifiers of key oncogenic proteins.^39^ Statins reduce systemic inflammatory mediators, such as interleukin 1‐beta and tumour necrosis factor,^40^, ^41^ as well as affecting epigenetic regulation via inhibition of deacetylation.^42^

LDL‐C‐independent effects are also reported for PCSK9 inhibitors,^43^ whose principal mode of action is the blockade of the circulating protein (PCSK9), which increases the number of hepatic LDLR and the plasma clearance of LDL‐C. The increased entry of LDL‐C may overburden the homeostatic mechanisms limiting cholesterol in the endoplasmic reticulum to 5% of total lipids.^44^

A recent systematic review of trials with the two commercially available monoclonal antibodies against PCSK9 provided no evidence of effect on cancer.^26^ In the present analysis, no differences in the risk for hormone‐sensitive cancers (both broad and strict definitions) were observed between treatment groups over a median observation period of approximately 2.5 years. As cholesterol is a precursor for steroid hormone production, low plasma cholesterol levels may impact the availability of sex hormones, which could potentially influence cancer incidence in certain malignancies.^6^, ^7^, ^8^ However, our results suggest that hormone‐dependent cancers are not particularly susceptible to changes in LDL‐C levels.

Though the protective effect of alirocumab in older patients may have been in part influenced by their higher event rates of cancer, it is tempting to speculate that, as we age, cancer development relies progressively more on plasma cholesterol as source of new membrane, and therefore becomes more responsive to therapies that lower LDL‐C to levels below the functional needs of cancer cells.

Limitations of this pooled, post hoc analysis include: (1) the limited exposure time (median 2.5 years; range up to 4 years); (2) trial designs, which substituted placebo for alirocumab in cases of sustained LDL‐C levels <15 mg/dL, thus precluding exploration of the effect of prolonged exposure to LDL‐C concentrations below that level; and (3) the sole use of investigator‐reported Medical Dictionary of Regulatory Activities to code cancer events. In addition, we recognise that there is an imbalance in the proportion of male (72%) and female (28%) participants among the pooled population used for this analysis. This imbalance could potentially be attributed to the patient enrolment criteria for entry into the cardiovascular trials that are included in this analysis. Moreover, a majority of patients in this pooled cohort analysis included participants from the ODYSSEY OUTCOMES trial of alirocumab in acute coronary syndrome, in which a large majority of participants were male. Furthermore, cardiovascular trials generally comprise a higher proportion of male participants due to the higher cardiovascular disease burden and given that females typically present with cardiovascular diseases at an older age compared to men.^45^, ^46^ Study strengths include: (1) the very large, pooled analysis from six placebo‐controlled clinical trials comprised of at‐risk populations; (2) baseline screening for trial participants that included systematically collected medical history information; (3) rate of cancer incidence in the placebo arm similar to age‐matched SEER*Explorer database^47^; and (4) frequent appraisals of medical status over the study period.

## 5 CONCLUSIONS

In this report, we show that substantial LDL‐C lowering with a PCSK9 inhibitor added to background statin therapy does not affect the risk of new or worsening cancer over the course of 2.5 years of median follow‐up. While no difference in risk of experiencing any type of neoplasm was observed for alirocumab versus placebo, a risk reduction in older patients (aged ≥64 years) was identified.

## AUTHOR CONTRIBUTIONS

**Kusha A. Mohammadi:** Conceptualization (equal); formal analysis (equal); methodology (equal); writing – original draft (equal); writing – review and editing (equal). **Taylor Brackin:** Conceptualization (equal); formal analysis (equal); methodology (equal); writing – original draft (equal); writing – review and editing (equal). **Gregory G. Schwartz:** Conceptualization (equal); formal analysis (equal); methodology (equal); writing – original draft (equal); writing – review and editing (equal). **Phillipe Gabriel Steg:** Conceptualization (equal); formal analysis (equal); methodology (equal); writing – original draft (equal); writing – review and editing (equal). **Michael Szarek:** Conceptualization (equal); formal analysis (equal); methodology (equal); writing – original draft (equal); writing – review and editing (equal). **Garen Manvelian:** Conceptualization (equal); formal analysis (equal); methodology (equal); writing – original draft (equal); writing – review and editing (equal). **Robert Pordy:** Conceptualization (equal); formal analysis (equal); methodology (equal); writing – original draft (equal); writing – review and editing (equal). **Sergio Fazio:** Conceptualization (equal); formal analysis (equal); methodology (equal); writing – original draft (equal); writing – review and editing (equal). **Gregory P. Geba:** Conceptualization (equal); formal analysis (equal); methodology (equal); writing – original draft (equal); writing – review and editing (equal).

## FUNDING INFORMATION

This analysis was funded by Regeneron Pharmaceuticals, Inc. The sponsor was involved in the study design and collection, analysis and interpretation of data, as well as data checking of information provided in the manuscript.

## CONFLICT OF INTEREST STATEMENT

KAH, GM, RP, SF, and GPG are all employees of and shareholders in Regeneron Pharmaceuticals, Inc. GGS reports research support to the University of Colorado from AstraZeneca, Resverlogix, Roche, Sanofi, and The Medicines Company; he is also coinventor of pending US patent 62/806,313 (‘Methods for Reducing Cardiovascular Risk’) assigned in full to the University of Colorado. PGS reports grants and nonfinancial support (cochair of the ODYSSEY OUTCOMES trial; as such, he received no personal fees, but his institution has received funding for the time he has devoted to trial coordination, and he has received support for travel related to trial meetings) from Sanofi; research grants and personal fees from Bayer (Steering Committee MARINER, grant for epidemiological study), Merck (speaker fees, grant for epidemiological studies), Sanofi (cochair of the ODYSSEY OUTCOMES trial; cochair of the SCORED trial; consulting, speaking), Servier (chair of the CLARIFY registry; grant for epidemiological research) and Amarin (executive steering committee for the REDUCE‐IT trial [Disease Reduction of Cardiovascular Events With Icosapent Ethyl–Intervention Trial]; consulting); and personal fees from Amgen, Bristol‐Myers Squibb, Boehringer Ingelheim, Pfizer, Idorsia, Myokardia, Novo Nordisk, Novartis, Regeneron Pharmaceuticals, Inc. and AstraZeneca. He also has a European application number/patent number, issued on October 26, 2016 (no. 15712241.7), for a method for reducing cardiovascular risk, all royalties assigned to Sanofi. MS receives salary support from CPC, a non‐profit academic research organisation affiliated with the University of Colorado that receives research grant/consulting funding from Abbott, Agios, Alexion Pharma, Alnylam, Amgen, Angionetics, ARCA Biopharma, Array, AstraZeneca, Atentiv, Audentes, Bayer, Better Therapeutics, Brigham and Women's Hospital, Bristol‐Myers Squibb, Cardiol Therapeutics, CellResearch, Cook Medical, Cook, CSL Behring, Eidos Therapeutics, EP Trading Co, Esperion Therapeutics, Everly Health, Faraday, Fortress Biotech, HDL Therapeutics, Heartflow, Hummingbird Bioscience, Insmed, Janssen, Kowa Research, Lexicon, Merck, MedPace, Medtronic, Moderna, Novate Medical, NovoNordisk, Pfizer, PhaseBio, PPD Development, Prairie Education and Research, Prothena Biosciences, Regeneron Pharmaceuticals, Inc., Regio Biosciences, Sanifit Therapeutics, Sanofi, Smith and Nephew, Stealth BioTherapeutics, University of Colorado, University of Pittsburgh, Worldwide Clinical Trials, Wraser and Yale Cardiovascular Research Group; has received fees for performing analyses, steering committee fees, and travel support from Sanofi and Regeneron Pharmaceuticals, Inc.; has received consulting fees from CiVi, Lexicon, Amarin, and Esperion; has received Data Safety and Monitoring Board membership fees from Resverlogix and Janssen; and is a member of *JACC* editorial board.

## ETHICS STATEMENT

All studies were conducted in accordance with the Declaration of Helsinki and the International Conference on Harmonisation Guidelines for Good Clinical Practice. All study protocols were approved by the appropriate institutional review board/ethics review committees, and patients provided written informed consent.

## Supporting information


Data S1.

## Data Availability

Qualified researchers may request access to study documents (including the clinical study report, study protocol with any amendments, blank case report form and statistical analysis plan) that support the methods and findings reported in this manuscript. Individual anonymised participant data will be considered for sharing once the product and indication has been approved by major health authorities (e.g., FDA, EMA, PMDA, etc.), if there is legal authority to share the data and there is not a reasonable likelihood of participant re‐identification. Submit requests to https://vivli.org/.
