# Supplementary material for: Effect of proprotein convertase subtilisin/kexin type 9 inhibition on cancer events: A pooled, post hoc, competing risk analysis of alirocumab clinical trials
Source: Cancer Med. 2023 Jul 17;12(16):16859–68. doi: 10.1002/cam4.6310 (PMC10501297; doi:10.1002/cam4.6310)
Supplement: Supplementary file 1 — Data S1. [file CAM4-12-16859-s001.docx]

# Supplementary information

Supplementary Table 1 Summary of patient demographic and baseline characteristics by any-neoplasm outcomes

| **Characteristic** | **Patients with any neoplasm  (n=969)** | **Patients without any neoplasm  (n=23** **101)** | **Overall  (N=24** **070)** |
| --- | --- | --- | --- |
| Age, median (IQR), years | 64.0 (57.0–70.0) | 59.0 (52.0–66.0) | 59.0 (52.0–66.0) |
| Sex, n (%) |  |  |  |
| Male | 709 (73) | 16 544 (72) | 17 253 (72) |
| Female | 260 (27) | 6557 (28) | 6817 (28) |
| Race, n (%) |  |  |  |
| White | 886 (91) | 18 648 (81) | 19 534 (81) |
| Black or African American | 31 (3.2) | 736 (3.2) | 767 (3.2) |
| Asian | 29 (3.0) | 2544 (11) | 2573 (11) |
| Other | 23 (2.4) | 1173 (5.1) | 1196 (5.0) |
| Weight, median (IQR), kg | 84.0 (75.0–94.8) | 82.0 (71.7–93.8) | 82.2 (71.9–93.9) |
| BMI, median (IQR) | 28.7 (26.1–31.8) | 28.3 (25.4–31.6) | 28.3 (25.5–31.6) |
| Smoking status, n (%) |  |  |  |
| Current | 177 (18) | 4374 (19) | 4551 (19) |
| Former or never | 621 (64) | 13 720 (59) | 14 341 (60) |
| Missing | 171 (18) | 5007 (22) | 5178 (22) |
| Drinking status, n (%) |  |  |  |
| Yes | 203 (21) | 3581 (16) | 3784 (16) |
| No | 595 (61) | 14 510 (63) | 15 105 (63) |
| Missing | 171 (18) | 5010 (22) | 5181 (22) |
| Hypertension status, n (%) |  |  |  |
| Yes | 581 (60) | 11 647 (50) | 12 228 (51) |
| No | 217 (22) | 6447 (28) | 6664 (28) |
| Missing | 171 (18) | 5007 (22) | 5178 (22) |
| History of any neoplasm diagnosis, n (%) | 75 (7.7) | 424 (1.8) | 499 (2.1) |
| Region of enrolment, n (%) |  |  |  |
| Asia | 27 (2.8) | 2299 (10) | 2326 (9.9) |
| East Europe | 211 (22) | 6590 (29) | 6801 (29) |
| North America | 286 (30) | 4167 (18) | 4453 (19) |
| Rest of world | 77 (8.1) | 1530 (6.8) | 1607 (6.8) |
| South America | 73 (7.7) | 2816 (12) | 2889 (12) |
| Western Europe | 275 (29) | 5132 (23) | 5407 (23) |
| Apolipoprotein A1, median (IQR), mg/dL | 136.0 (121.0–152.0) | 134.0 (119.0–151.0) | 134.0 (119.0–151.0) |
| Lipoprotein(a), median (IQR), mg/dL | 24.4 (7.8–64.7) | 21.0 (6.7–60.1) | 21.1 (6.8–60.3) |
| Total cholesterol, median (IQR), mg/dL | 162.0 (144.0–187.0) | 165.6 (145.6–193.8) | 165.6 (145.6– 193.4) |
| Low-density lipoprotein cholesterol, median (IQR), mg/dL | 88.0 (75.0–108.9) | 90.7 (75.7–112.7) | 90.3 (75.7–112.7) |
| High-density lipoprotein cholesterol, median (IQR), mg/dL | 43.6 (37.1–52.0) | 43.2 (37.0–51.4) | 43.2 (37.0–51.4) |
| Triglycerides, median (IQR), mg/dL | 129.2 (89.4–181.0) | 131.0 (95.6–185.0) | 131.0 (95.0–184.1) |
| Haemoglobin A1c, median (IQR), % | 5.8 (5.5–6.3) | 5.9 (5.5–6.4) | 5.8 (5.5– 6.4) |

BMI, body mass index; IQR, interquartile range.

Any neoplasm is defined as benign, unspecified, or malignant neoplasms.

Supplementary Table 2 Summary of cancer incidence during the treatment-emergent adverse event period by treatment group and study

| **Type of event** | **Study** | **Placebo** | | | **Alirocumab** | | |
| --- | --- | --- | --- | --- | --- | --- | --- |
|  |  | **No. of patients** | **No. of events** | **n (%)** | **No. of patients** | **No. of events** | **n (%)** |
| Any neoplasm | Combined | 11 537 | 732 | 497 (4.31) | 12 533 | 638 | 472 (3.77) |
|  | EFC11568 | 107 | 7 | 5 (4.67) | 207 | 5 | 5 (2.42) |
|  | EFC11570 | 9441 | 622 | 406 (4.30) | 9451 | 547 | 392 (4.15) |
|  | EFC12732 | 35 | 1 | 1 (2.86) | 72 | 0 | 0 |
|  | LTS11717 | 788 | 41 | 32 (4.06) | 1550 | 52 | 43 (2.77) |
|  | R727-CL-1112 | 81 | 0 | 0 | 167 | 5 | 5 (2.99) |
|  | R727-CL-1532 | 1085 | 61 | 53 (4.88) | 1086 | 29 | 27 (2.49) |
| Malignant neoplasm | Combined | 11 537 | 525 | 343 (2.97) | 12 533 | 436 | 318 (2.54) |
|  | EFC11568 | 107 | 4 | 2 (1.87) | 207 | 2 | 2 (0.97) |
|  | EFC11570 | 9441 | 448 | 282 (2.99) | 9451 | 376 | 266 (2.81) |
|  | EFC12732 | 35 | 0 | 0 | 72 | 0 | 0 |
|  | LTS11717 | 788 | 32 | 24 (3.05) | 1550 | 36 | 28 (1.81) |
|  | R727-CL-1112 | 81 | 0 | 0 | 167 | 1 | 1 (0.60) |
|  | R727-CL-1532 | 1085 | 41 | 35 (3.23) | 1086 | 21 | 21 (1.93) |
| Hormone-sensitive – broad | Combined | 11 537 | 104 | 83 (0.72) | 12 533 | 95 | 75 (0.60) |
|  | EFC11568 | 107 | 0 | 0 | 207 | 0 | 0 |
|  | EFC11570 | 9441 | 82 | 62 (0.66) | 9451 | 85 | 65 (0.69) |
|  | EFC12732 | 35 | 0 | 0 | 72 | 0 | 0 |
|  | LTS11717 | 788 | 9 | 8 (1.02) | 1550 | 5 | 5 (0.32) |
|  | R727-CL-1112 | 81 | 0 | 0 | 167 | 0 | 0 |
|  | R727-CL-1532 | 1085 | 13 | 13 (1.20) | 1086 | 5 | 5 (0.46) |
| Hormone-sensitive – strict | Combined | 11 537 | 86 | 71 (0.62) | 12 533 | 81 | 65 (0.52) |
|  | EFC11568 | 107 | 0 | 0 | 207 | 0 | 0 |
|  | EFC11570 | 9441 | 68 | 53 (0.56) | 9451 | 71 | 55 (0.58) |
|  | EFC12732 | 35 | 0 | 0 | 72 | 0 | 0 |
|  | LTS11717 | 788 | 5 | 5 (0.63) | 1550 | 5 | 5 (0.32) |
|  | R727-CL-1112 | 81 | 0 | 0 | 167 | 0 | 0 |
|  | R727-CL-1532 | 1085 | 13 | 13 (1.20) | 1086 | 5 | 5 (0.46) |

Any neoplasm is defined as benign, unspecified, or malignant neoplasms. Malignant neoplasm contain only identified malignant neoplasms. Hormone sensitive – broad includes breast, prostate, uterine, ovarian, pancreas, gallbladder, liver, colorectal, and gastrointestinal cancers. Hormone sensitive – strict includes breast, prostate, uterine, and ovarian cancers. EFC11568: ODYSSEY COMBO I (NCT01644175); EFC115070: ODYSSEY OUTCOMES (NCT01663402); EFC12732: ODYSSEY OLE (NCT01954394); LTS11717: ODYSSEY LONG TERM (NCT01507831); R727-CL-1112: ODYSSEY FH II (NCT01709500); R727-CL-1532: Neurocognitive (NCT02957682).

Supplementary Table 3 Breakdown of outcome components during the treatment-emergent adverse event period by treatment group

| **Outcome** | **Composite Component, n/N (%)** | **Placebo (n=11 537)** | **Alirocumab (n=12 533)** |
| --- | --- | --- | --- |
| First any neoplasm | Any | 497/11 537 (4.31) | 472/12 533 (3.77) |
|  | Benign or unspecified neoplasm | 161/497 (32.39) | 162/472 (34.32) |
|  | Not hormone-sensitive malignant neoplasm | 241/497 (48.49) | 228/472 (48.31) |
|  | Hormone-sensitive – strict | 67/497 (13.48) | 63/472 (13.35) |
|  | Hormone-sensitive – broad | 11/497 (2.21) | 8/472 (1.69) |
|  | Metastatic cancers | 17/497 (3.42) | 11/472 (2.33) |
| First malignant neoplasm | Any | 343/11 537 (2.97) | 318/12 533 (2.54) |
|  | Not hormone-sensitive malignant neoplasm | 248/343 (72.30) | 233/318 (73.27) |
|  | Hormone-sensitive – strict | 67/343 (19.53) | 65/318 (20.44) |
|  | Hormone-sensitive – broad | 11/343 (3.21) | 9/318 (2.83) |
|  | Metastatic cancers | 17/343 (4.96) | 11/318 (3.46) |
| First hormone-sensitive – broad | Any | 83/11 537 (0.72) | 75/12 533 (0.60) |
|  | Hormone-sensitive – strict | 71/83 (85.54) | 65/75 (86.67) |
|  | Hormone-sensitive – broad | 12/83 (14.46) | 10/75 (13.33) |
| First hormone-sensitive – strict | Any | 71/11 537 (0.62) | 65/12 533 (0.52) |
|  | Hormone-sensitive – strict | 71/71 (100.0) | 65/65 (100.0) |

Any neoplasm was defined as benign, unspecified, or malignant neoplasms. Malignant neoplasm contains only identified malignant neoplasms. Hormone sensitive – broad includes breast, prostate, uterine, ovarian, pancreas, gallbladder, liver, colorectal, and gastrointestinal cancers. Hormone sensitive – strict includes breast, prostate, uterine, and ovarian cancers.

Supplementary Table 4. Sensitivity analyses for time to first cancer outcome during the treatment-emergent adverse event period

| **Hazard model type and outcome** | **Alirocumab versus placebo** | | |
| --- | --- | --- | --- |
|  | **HR (95% CI)** | **Model-based p value** | **Log rank/Gray’s test p value** |
| Proportional | | | |
| First any neoplasm | 0.93 (0.82–1.06) | 0.27 | 0.27 |
| First malignant neoplasm | 0.91 (0.79–1.07) | 0.25 | 0.25 |
| First hormone-sensitive – broad | 0.89 (0.65–1.22) | 0.48 | 0.48 |
| First hormone-sensitive – strict | 0.90 (0.65–1.26) | 0.55 | 0.55 |
| Cause-specific | | | |
| First any neoplasm | 0.93 (0.82–1.06) | 0.27 |  |
| First malignant neoplasm | 0.91 (0.79–1.07) | 0.25 |  |
| First hormone-sensitive – broad | 0.89 (0.65–1.22) | 0.48 |  |
| First hormone-sensitive – strict | 0.90 (0.65–1.27) | 0.55 |  |

Any neoplasm was defined as benign, unspecified, or malignant neoplasms. Malignant neoplasm contains only identified malignant neoplasms. Hormone sensitive - broad includes breast, prostate, uterine, ovarian, pancreas, gallbladder, liver, colorectal, and gastrointestinal cancers. Hormone sensitive – strict includes breast, prostate, uterine, and ovarian cancers. Hazard ratio (95% CI) is based on an associated hazard model type with randomised treatment as the only model term.

CI, confidence interval; HR, hazards ratio.

Supplementary Figure 1 Alirocumab clinical trials included in this analysis


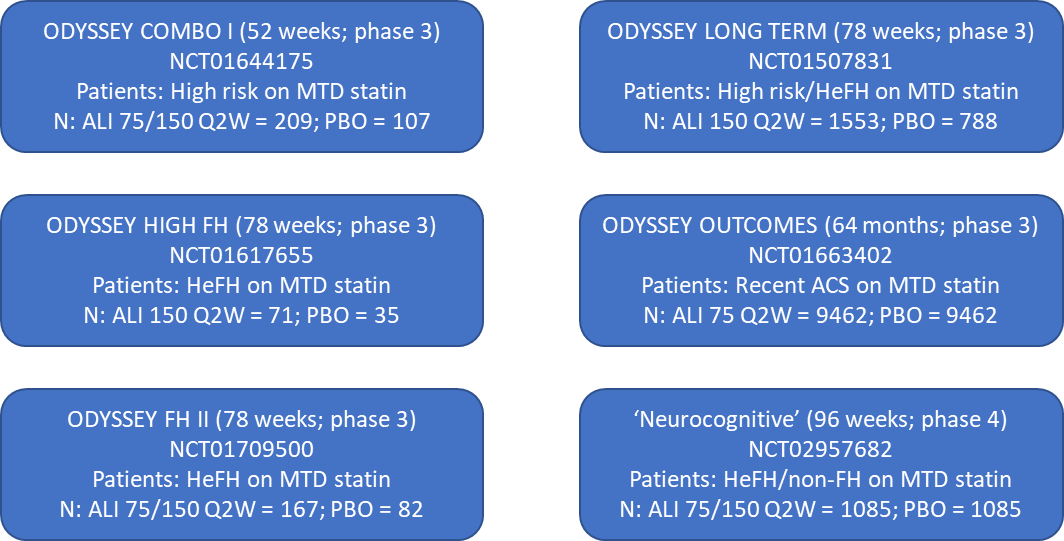


ALI, alirocumab; FH, familial hypercholesterolemia; HeFH, heterozygous familial hypercholesterolaemia; LDL-C, low-density lipoprotein cholesterol; MTD, maximum-tolerated dose; PBO, placebo; Q2W, every 2 weeks.

The figure shows the study name, double-blind trial duration, phase, clinicaltrials.gov identifier, patient disease background, patient background statin usage, and number of patients randomised to the ALI and PBO groups.

ALI 75/150 Q2W indicates where the starting dose of ALI 75 mg Q2W could be increased to 150 mg Q2W at week 12 if pre-specified LDL-C levels were not achieved by week 8.

Supplementary Figure 2 Histogram for the duration of the treatment-emergent adverse event period


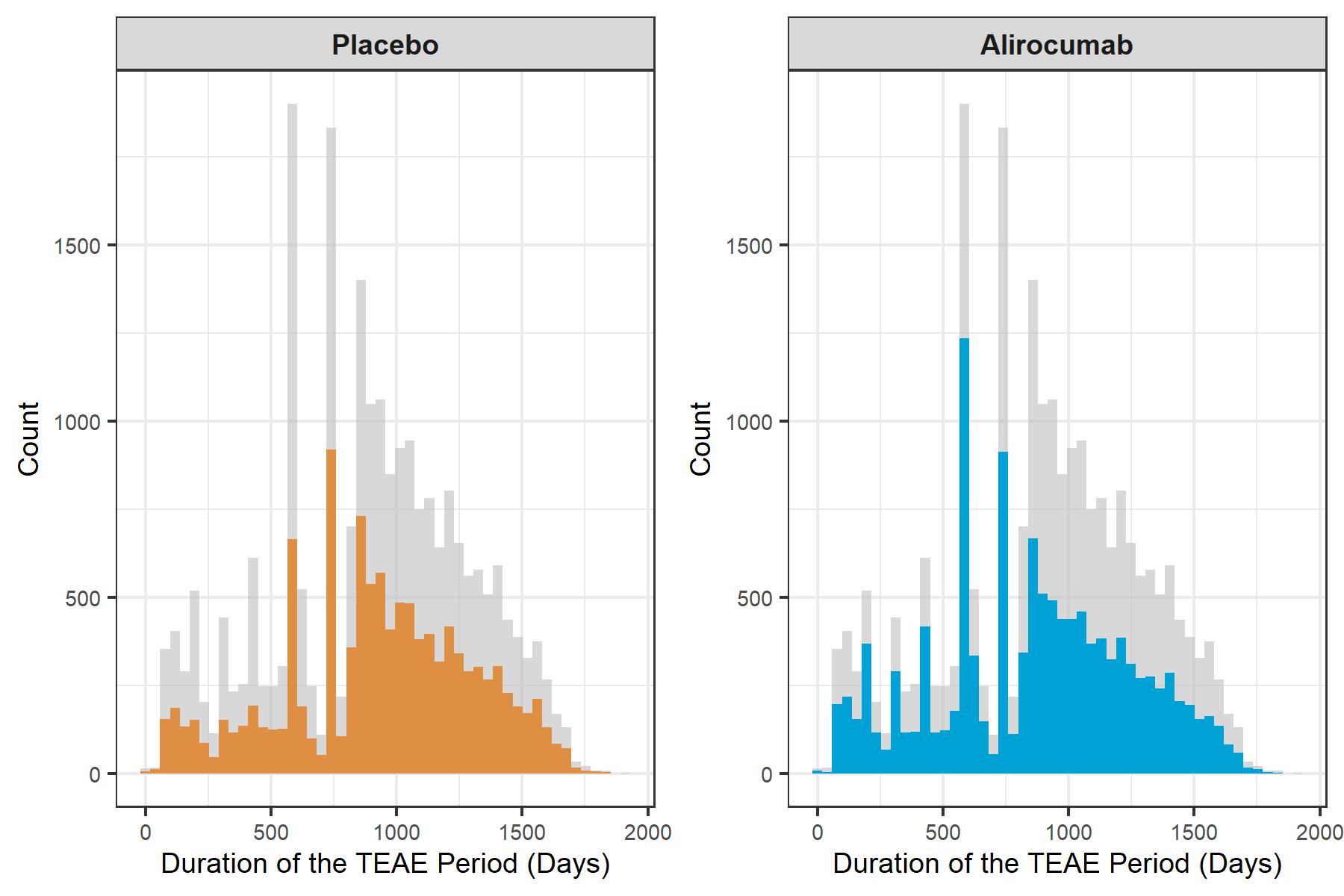


TEAE, treatment-emergent adverse event.

The grey bars in the background of each grid represent the overall distribution for the duration of the TEAE period.

Supplementary Figure 3 Cumulative incidence using competing risk for time to first malignant neoplasm during (A) the treatment-emergent adverse event period in the whole population, and among those (B) older or (C) younger than the median age of patients with cancer (64 years)

**(A)**


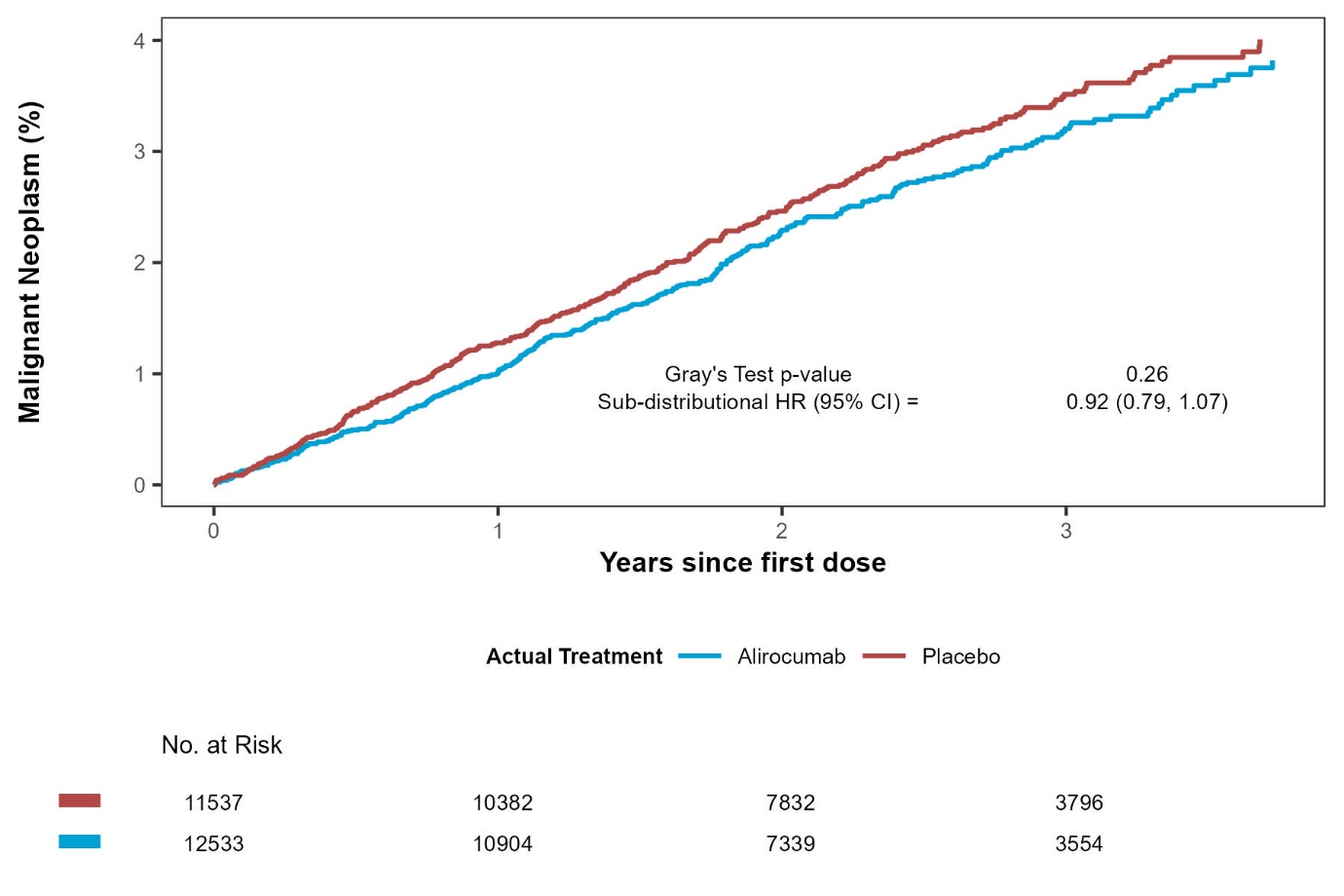


**(B)**


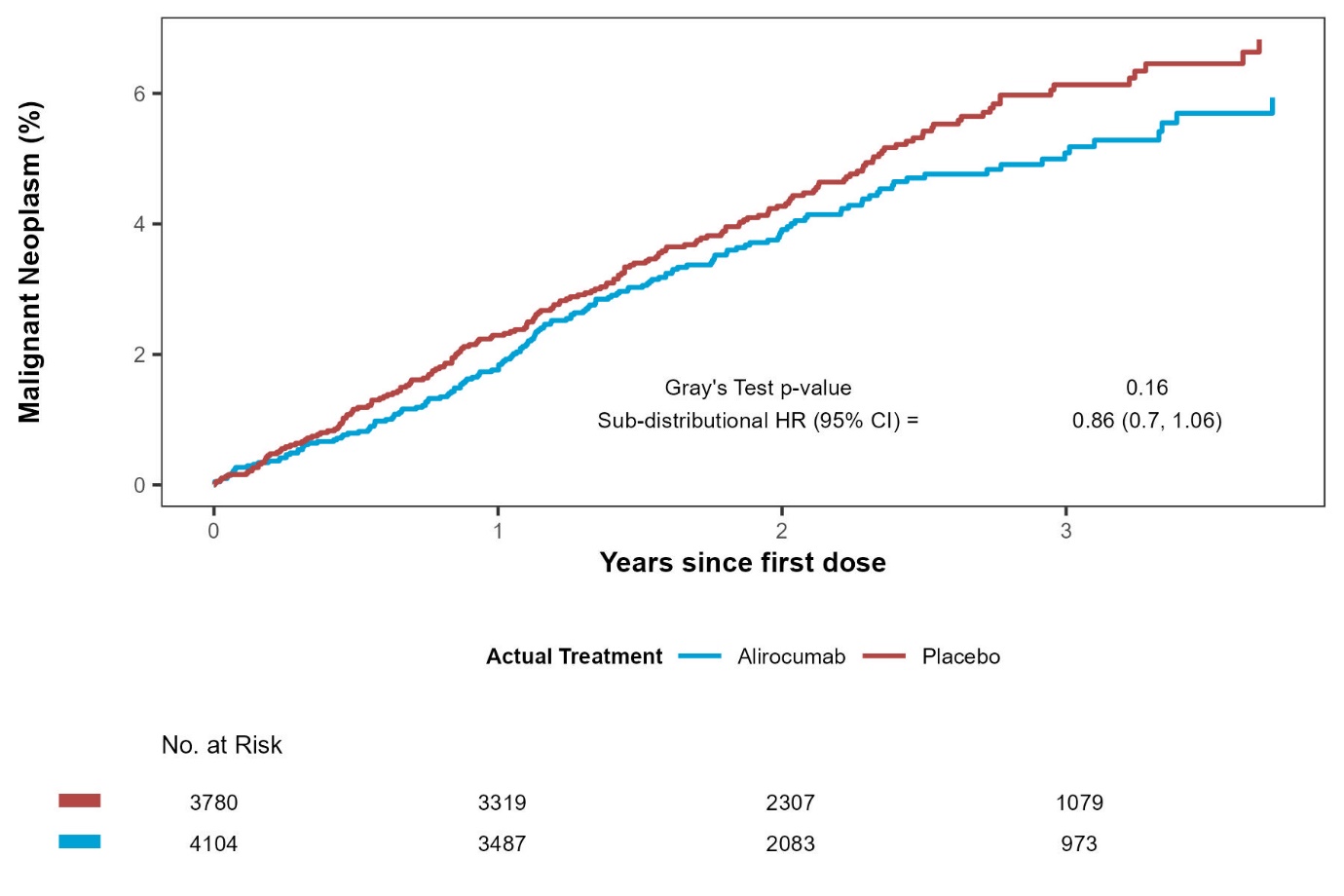


**(C)**


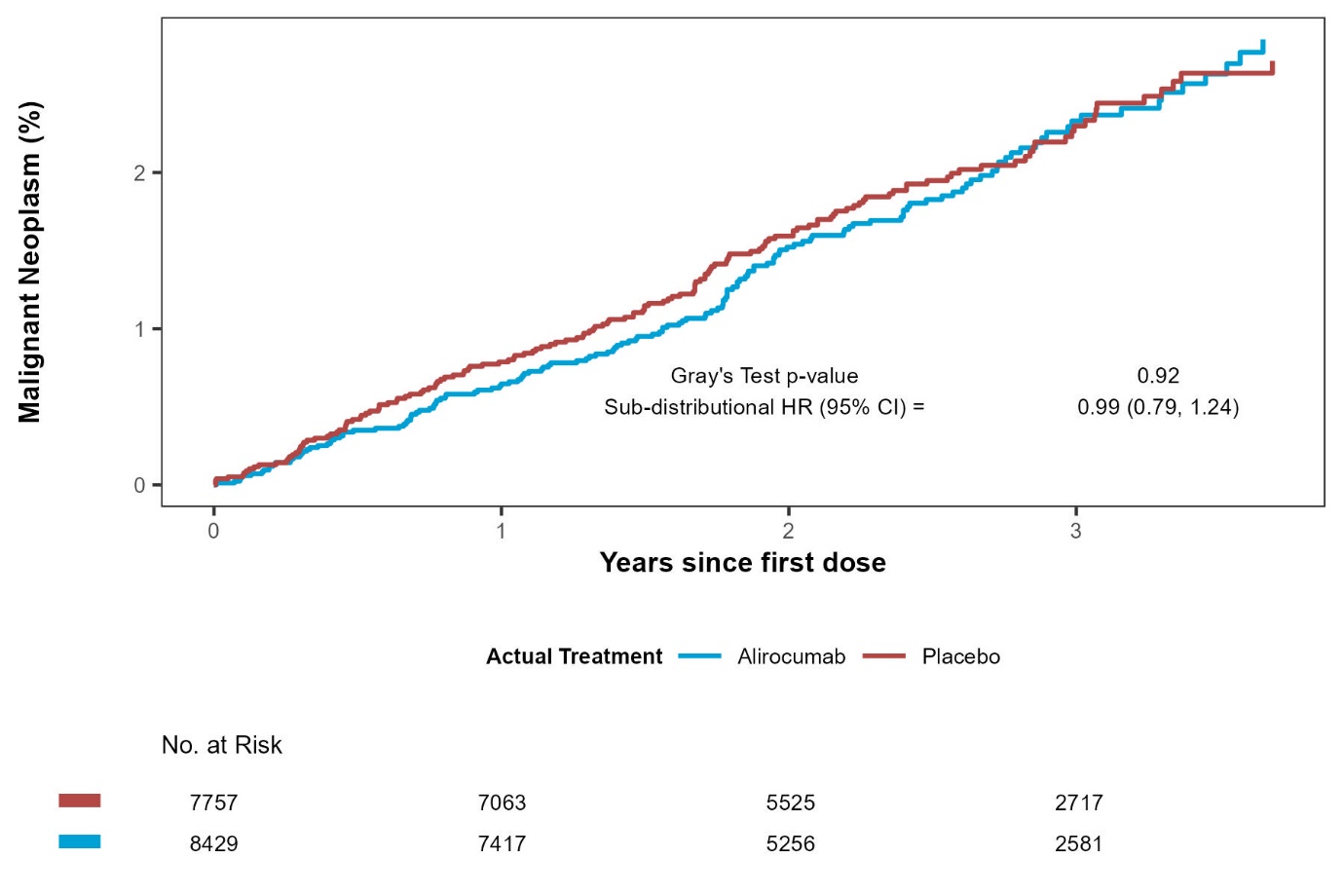


CI, confidence interval; HR, hazard ratio.

Supplementary Figure 4 Cumulative incidence using competing risk for time to first hormone-sensitive – broad neoplasm during the treatment-emergent adverse event period in (A) the whole population, and among those (B) older or (C) younger than the median age of patients with cancer (64 years)

**(A)**


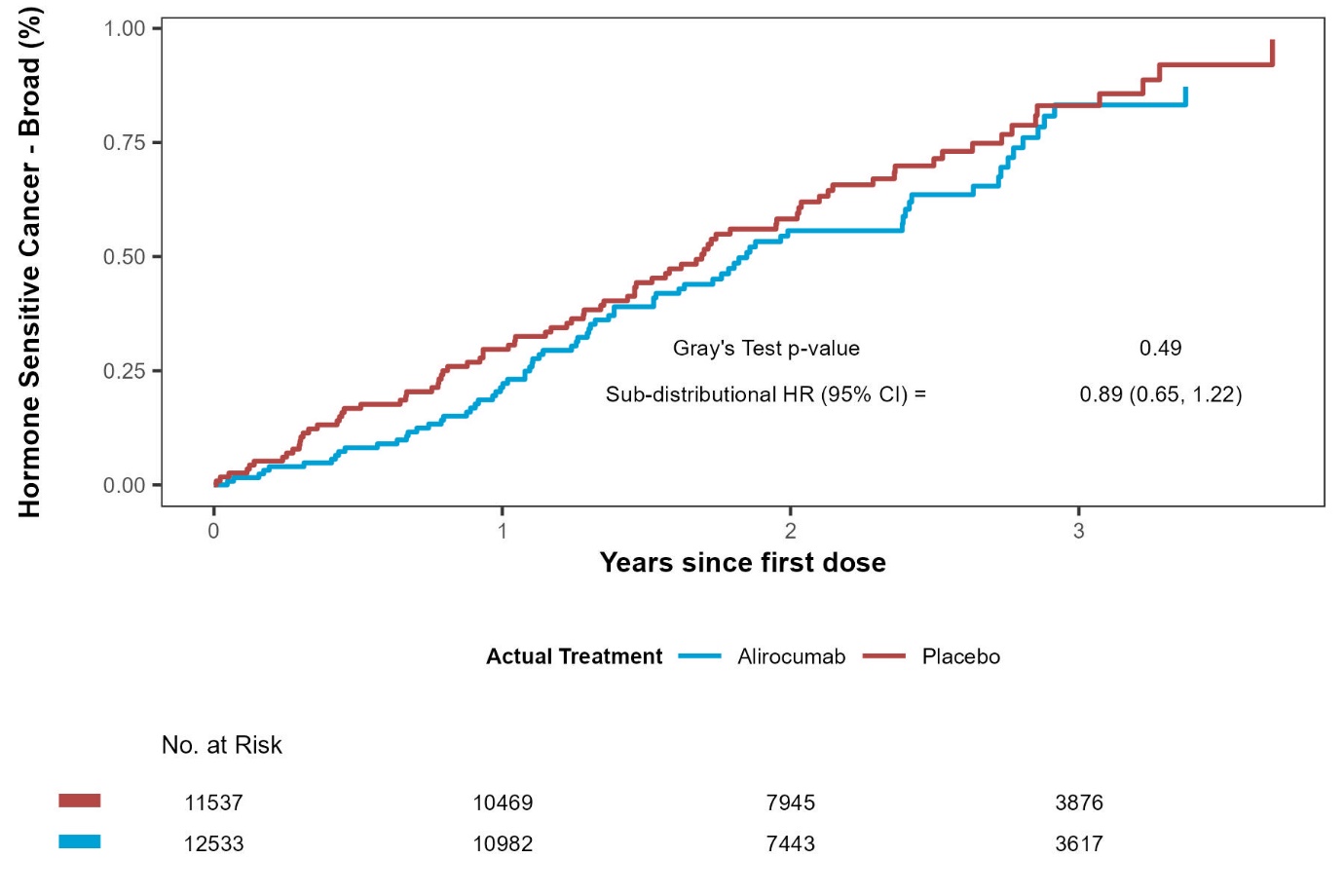


**(B)**


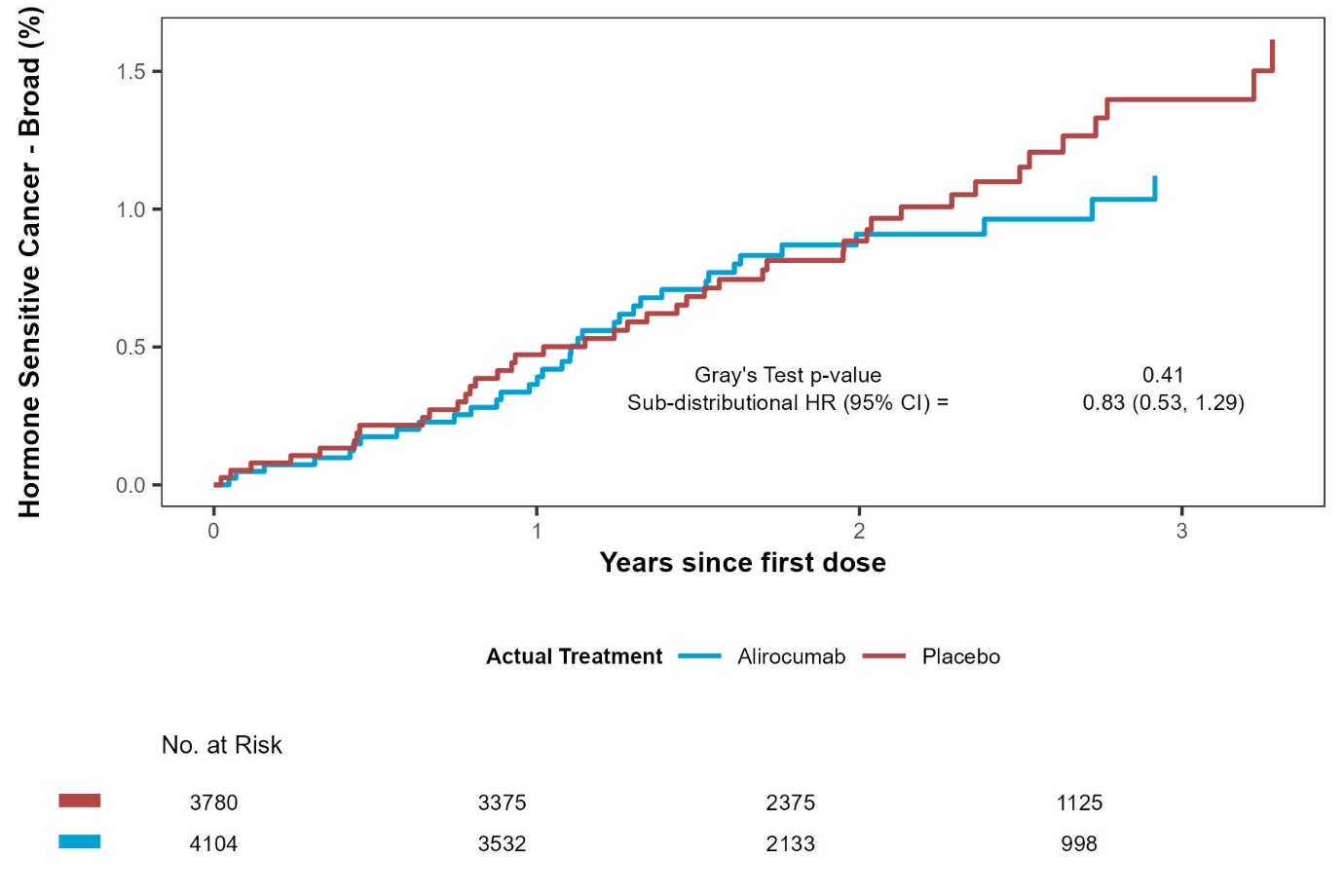


**(C)**


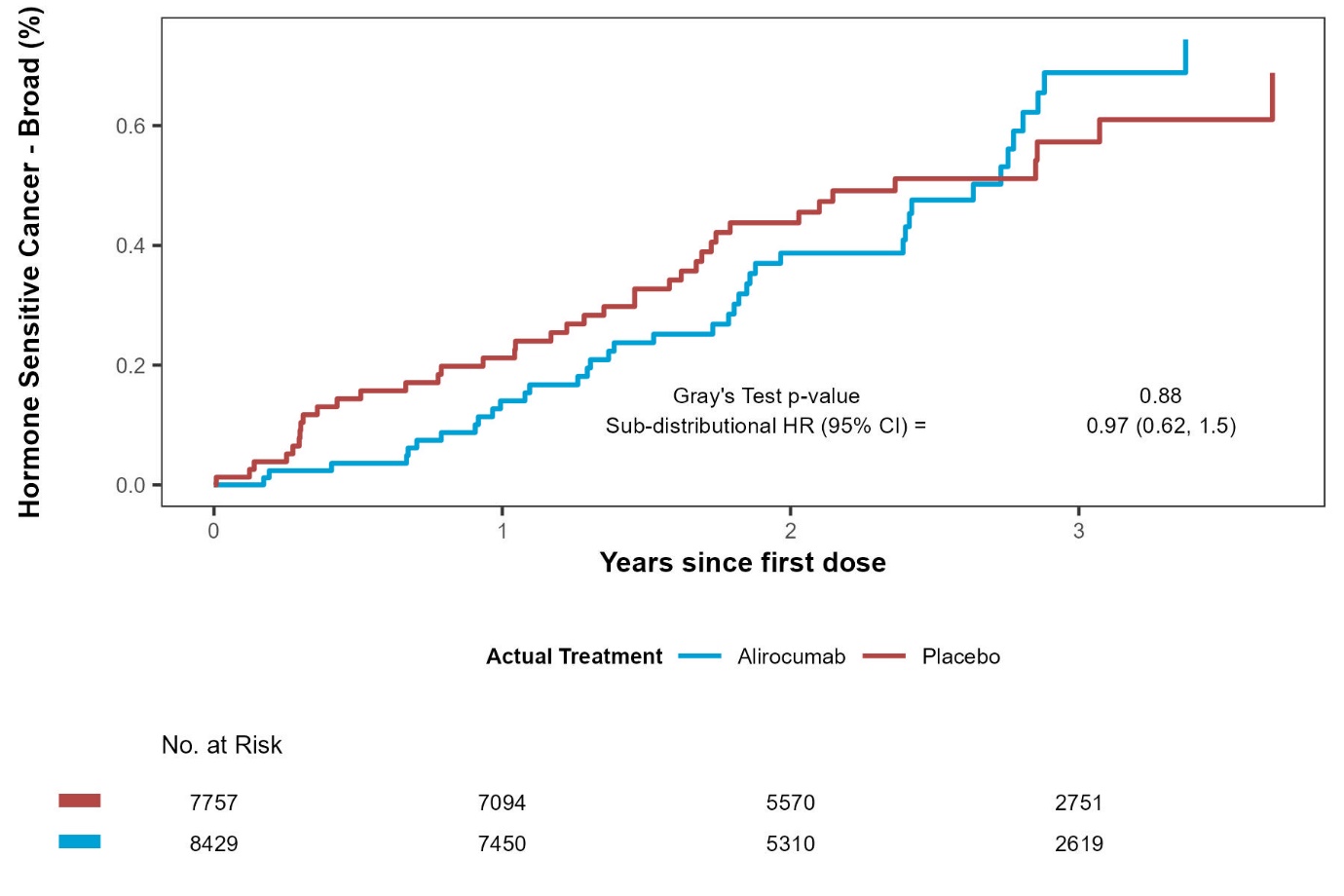


CI, confidence interval; HR, hazard ratio.

Supplementary Figure 5 Cumulative incidence using competing risk for time to first hormone-sensitive – strict neoplasm during the treatment-emergent adverse event period in (A) the whole population, and among those (B) older or (C) younger than the median age of patients with cancer (64 years)

**(A)**


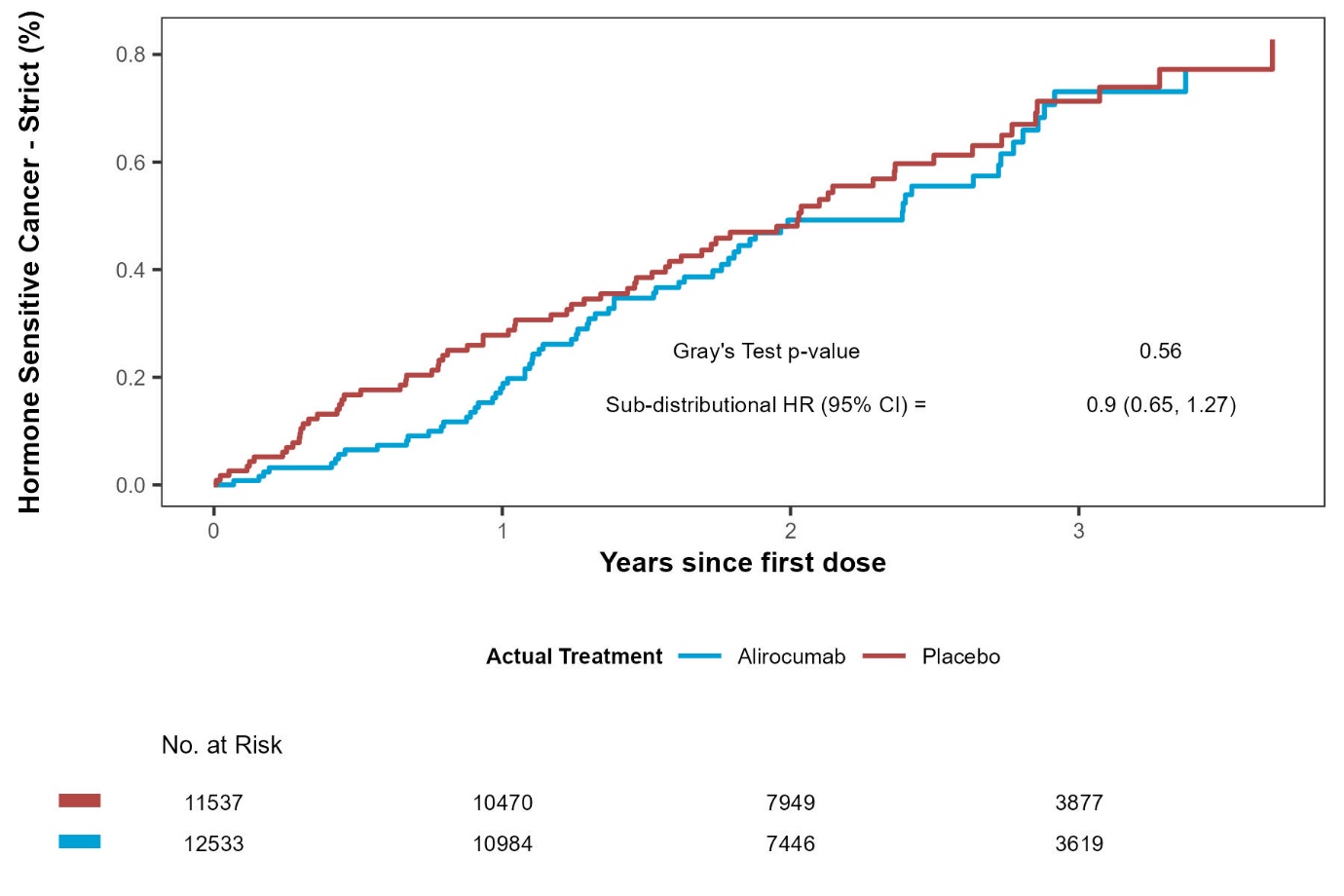


**(B)**


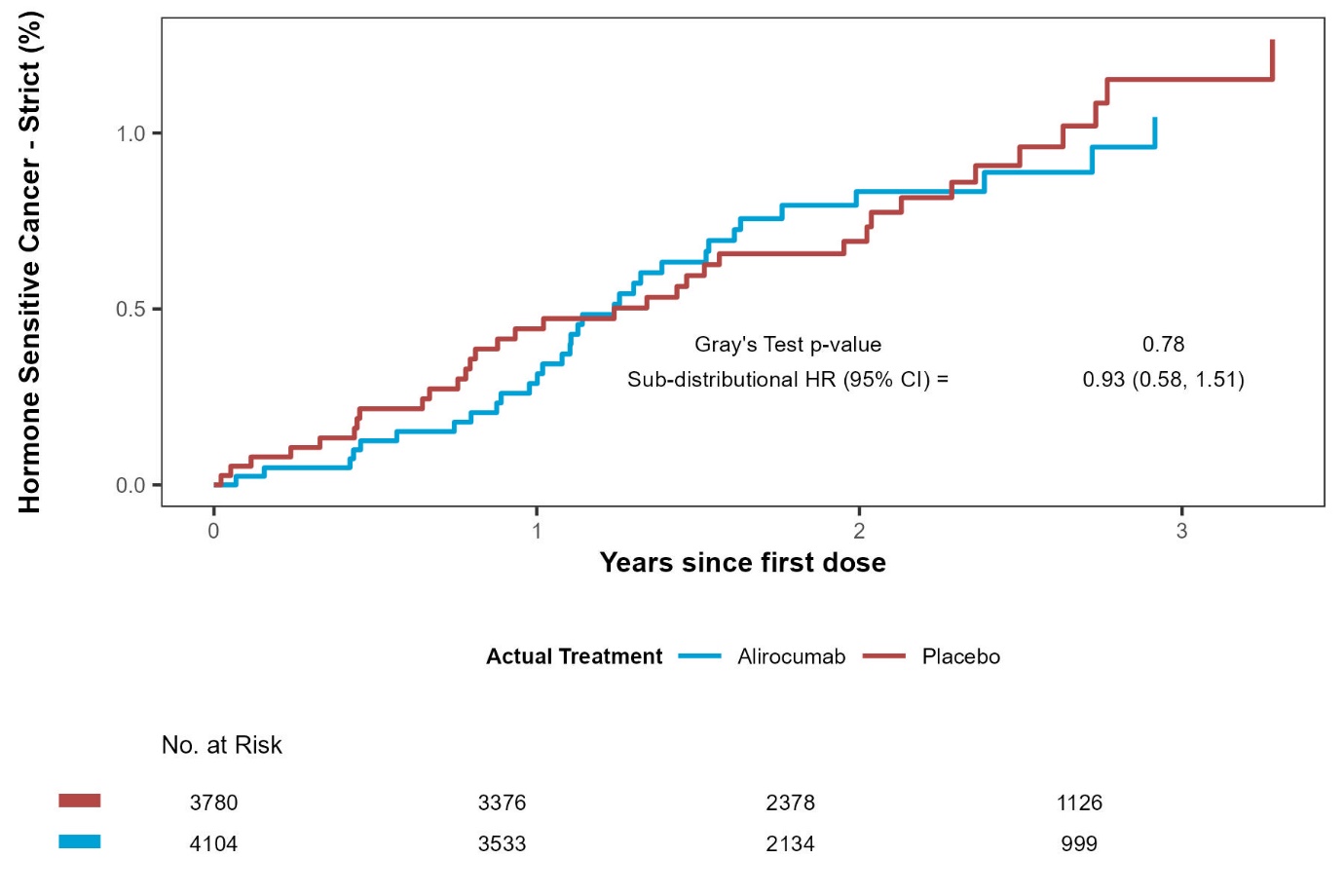


**(C)**


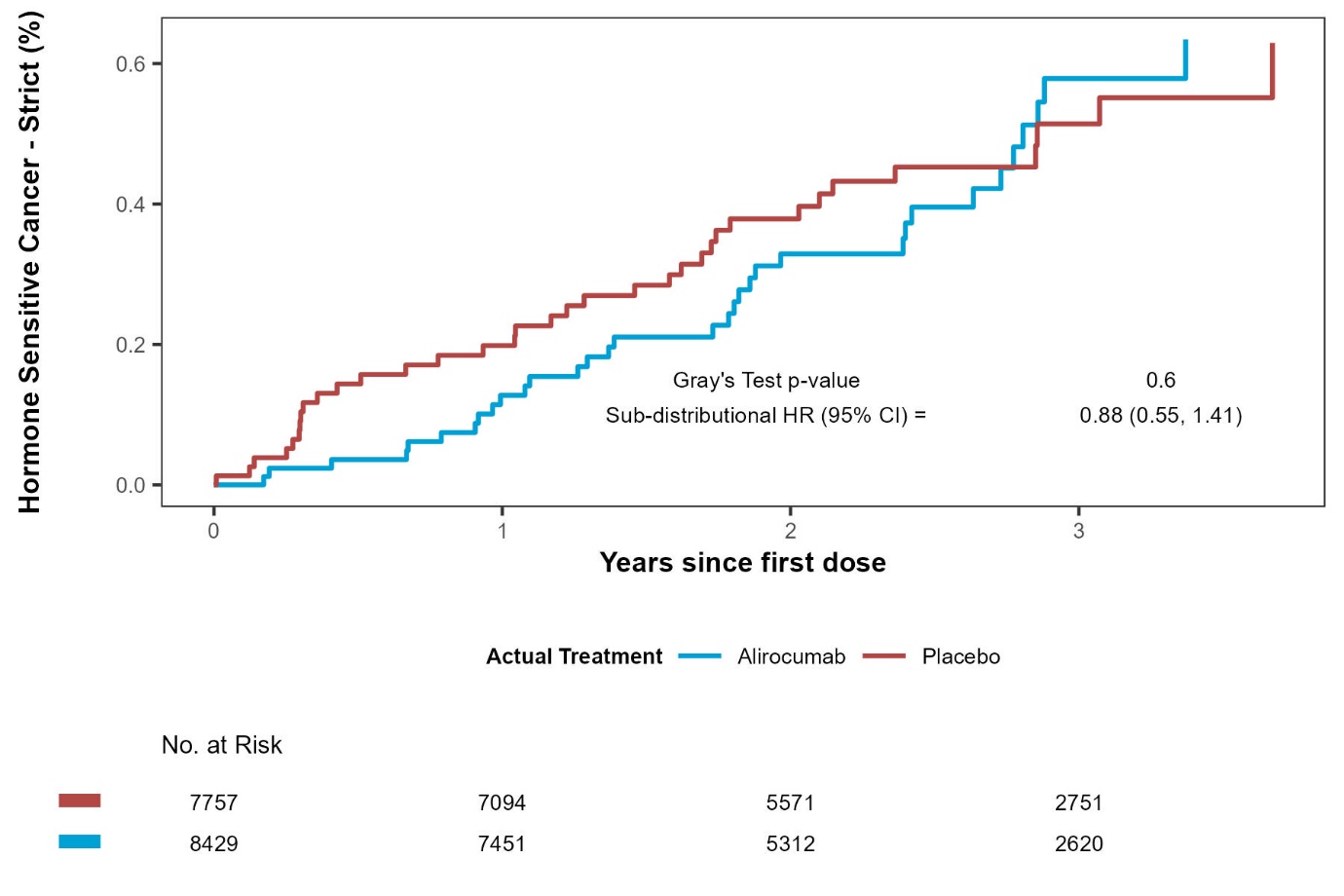


CI, confidence interval; HR, hazard ratio.
